# Nanoscale structural characterization of epitaxial graphene grown on off-axis 4H-SiC (0001)

**DOI:** 10.1186/1556-276X-6-269

**Published:** 2011-03-29

**Authors:** Carmelo Vecchio, Sushant Sonde, Corrado Bongiorno, Martin Rambach, Rositza Yakimova, Vito Raineri, Filippo Giannazzo

**Affiliations:** 1 CNR-IMM, Strada VIII, 5, Catania 95121, Italy; 2 Scuola Superiore di Catania, Via San Nullo, 5/i, Catania 95123, Italy; 3 Centrotherm Thermal Solutions GmbH + Co. KG, Johannes-Schmid-Straße 8, Blaubeuren 89143, Germany; 4 IFM, Linkoping University, Linkoping, Sweden

## Abstract

In this work, we present a nanometer resolution structural characterization of epitaxial graphene (EG) layers grown on 4H-SiC (0001) 8° off-axis, by annealing in inert gas ambient (Ar) in a wide temperature range (*T*_gr _from 1600 to 2000°C). For all the considered growth temperatures, few layers of graphene (FLG) conformally covering the 100 to 200-nm wide terraces of the SiC surface have been observed by high-resolution cross-sectional transmission electron microscopy (HR-XTEM). Tapping mode atomic force microscopy (t-AFM) showed the formation of wrinkles with approx. 1 to 2 nm height and 10 to 20 nm width in the FLG film, as a result of the release of the compressive strain, which builds up in FLG during the sample cooling due to the thermal expansion coefficients mismatch between graphene and SiC. While for EG grown on on-axis 4H-SiC an isotropic mesh-like network of wrinkles interconnected into nodes is commonly reported, in the present case of a vicinal SiC surface, wrinkles are preferentially oriented in the direction perpendicular to the step edges of the SiC terraces. For each *T*_gr_, the number of graphene layers was determined on very small sample areas by HR-XTEM and, with high statistics and on several sample positions, by measuring the depth of selectively etched trenches in FLG by t-AFM. Both the density of wrinkles and the number of graphene layers are found to increase almost linearly as a function of the growth temperature in the considered temperature range.

## Introduction

Graphene has attracted the interest of the scientific community due to its excellent electronic transport properties [1,2], which make it a promising material for ultra-fast electronics operating in the 100 GHz to THz frequencies [3]. Graphene was first obtained by mechanical exfoliation of highly oriented pyrolithic graphite (HOPG) [1]. This is still the method of choice for basic studies on this material, because it produces high-crystalline quality graphene samples, which can be deposited on several substrates [4,5]. However, the size of these flakes and the control of location on the substrate are not adequate for development of integrated circuit technologies. One major challenge to realize graphene electronics is to be able to produce large area and laterally uniform films. To date, the main routes toward large area graphene production include chemical vapor deposition (CVD) on metals [6], and controlled graphitization of hexagonal SiC by high-temperature thermal processes [7].

The latter method is the strongest candidate for graphene based electronics, owing to the fact that few layers of graphene (FLG) can be grown on a semiconductor substrate without any need to transfer (as is the case with CVD graphene on metal substrates). Graphene such produced is known as epitaxial graphene (EG). Graphene growth during annealing of SiC relies on the interplay of two different mechanisms: (i) the preferential Si sublimation from SiC surface, which leaves an excess of C atoms in the near surface region, and (ii) the diffusion of these C atoms on the SiC surface and their reorganization in the two-dimensional graphene lattice structure. Both mechanisms depend in a complex way both on the annealing conditions (temperature *T*_gr_, ramp rate, pressure in the chamber) and on the initial surface conditions of SiC wafers, e.g., the termination (Si- or C-face), the miscut angle, the defectivity. It has been shown that the rate of Si evaporation can be strongly reduced by performing the annealing in inert gas ambient at atmospheric pressure instead than in vacuum [8,9]. This results in the possibility to raise the growth temperature, from typical values of *T*_gr _~1300°C in vacuum to *T*_gr _> 1600°C at atmospheric pressure [8]. This has, in turns, beneficial effects on the crystalline quality of the EG layers, since at higher temperature, a higher diffusivity of the C atoms on the surface is achieved. It has been also observed that graphene growth starts from the kinks of terraces on the SiC surface or from defects (e.g., pits or threading dislocations) [10]. This means that, for fixed annealing conditions, a higher growth rate is expected on vicinal, i.e., off-axis, SiC surfaces than on on-axis ones, because the spacing between terraces kinks is reduced. Most of the studies on EG growth reported in the literature were carried out on on-axis hexagonal SiC substrates. However, off-axis 4H-SiC (0001) substrates with lowly doped epilayers are the standard platform for SiC technology. Hence, a detailed investigation on graphene growth on such substrates is mandatory, in the perspective of integrating future graphene electronics with current SiC technology.

In this work, we present a structural characterization of EG growth on the Si face of 8° off-axis 4H-SiC [11] by annealing in inert gas (Ar) ambient in a wide range of temperatures (*T*_gr _from 1600 to 2000°C). The modifications in the surface morphology after thermal treatments at the different temperatures were studied by tapping mode atomic force microscopy (t-AFM), showing the increase in the density of peculiar corrugations (wrinkles) in the EG multilayers with the growth temperature. High-resolution cross-sectional transmission electron microscopy (HRXTEM) was used to determine the number of graphene layers on very small sample areas. An alternative method to estimate the EG thickness on larger areas, based on etching out trenches selectively in graphitized SiC and measurements of the depth of those trenches by AFM, is also presented. The accuracy of this method is discussed.

## Experimental details

Graphene growth was carried out in argon (Ar) ambient in a commercial furnace by Centrotherm Thermal Solutions GmbH + Co. KG, Blaubeuren, Germany at temperatures *T*_gr _from 1600 to 1700°C, or in an inductively heated reactor at *T*_gr _= 2000°C. The substrates used for graphene growth were lowly doped (approx. 10^14 ^cm^-3^) epitaxially grown SiC layers on top of highly doped, 8° off-axis 4H-SiC (0001).

t-AFM measurements were carried out with Veeco DI3100 atomic force microscope fitted with Nanoscope V controller. Commercial silicon probes with spring constant *k *= 20 to 80 N/m, oscillation frequency 332 to 375 kHz in high-amplitude mode were employed.

Cross-sectional observations of the few layers of graphene grown on SiC were carried out using a JEOL JEM 2010F transmission electron microscope with a Schottky field emission gun operating at an acceleration voltage of 200 kV. Thin specimens for TEM observation were prepared by Ar-ion thinning by the following procedure: the graphene-on-SiC samples were mechanically ground to 70 μm thick, and the center of the sample was thinned by dimple grinding, followed by Ar-ion beam thinning under a low angle of 8° at 5 kV.

HRXTEM images allow an extremely precise determination of the number of grown layers, but on very small areas. An alternative method to determine the thickness of the grown FLG film on larger areas has been also developed. Stripes were patterned onto graphitized SiC substrates by optical lithography. Graphene was then selectively removed from these stripes with an O_2 _plasma (25 sccm) treatment for 16 min in an Roth & Rau Microsys 400 ICP-RIE (inductively coupled plasma) equipment. The step height between etched and not etched regions was determined by t-AFM. Before each t-AFM map, we acquired amplitude-distance curves both on not-etched and etched regions and the amplitude setpoint as well as the free amplitude were chosen accordingly, in order to operate in the repulsive interaction regime [12] on both surfaces.

The etch rates of graphene and bare SiC were calibrated independently. To this aim, graphene flakes were exfoliated from HOPG on SiC and their thickness was measured after different etching times by AFM. A patterned virgin SiC sample was etched under identical conditions and the etched depth was also measured by AFM. For the used plasma conditions, the etch rate of graphene is much higher than that of bare SiC.

## Results and discussion

**Figure 1:**
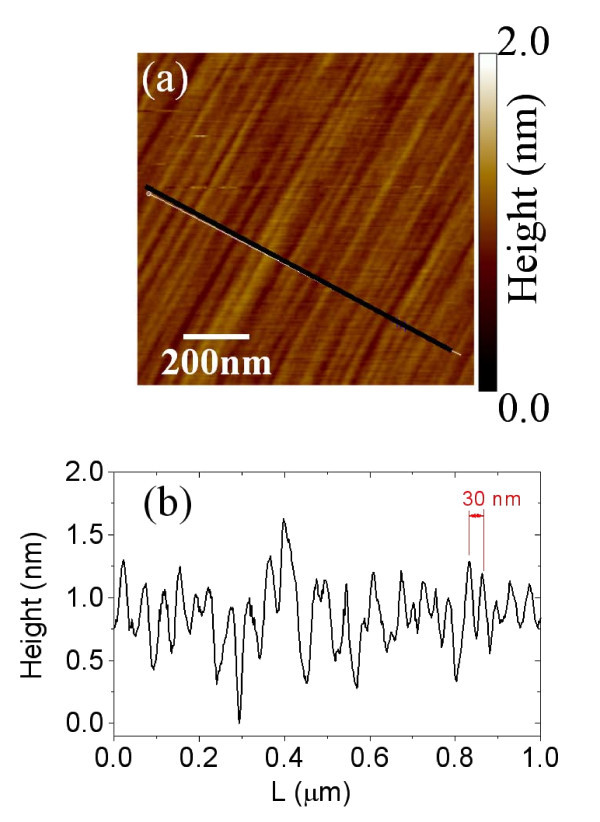
**Morphology of the virgin 4H-SiC (0001), 8° off-axis surface**. AFM image (a) and linescan in the direction orthogonal to the steps (b).

A morphological AFM image of the virgin 4H-SiC (0001) surface is reported in Figure 1a, showing the parallel terraces oriented in the < 00-10 > direction. The mean width of the terraces (approx. 30 nm) can be deduced from the linescan in Figure 1b.

A root mean square (RMS) roughness of 0.2 nm is obtained from this surface analysis.

**Figure 2:**
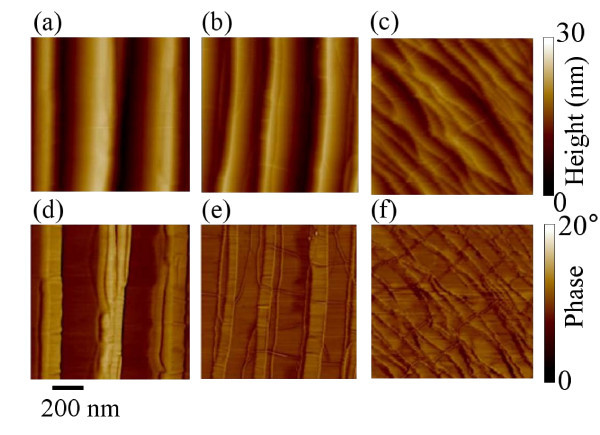
**AFM morphology and phase of SiC samples annealed at different temperatures**. Surface morphology for the samples annealed at 1600 (a), 1700 (b) and 2000°C (c), and corresponding phase maps on the same samples ((d), (e) and (f)).

**Figure 3:**
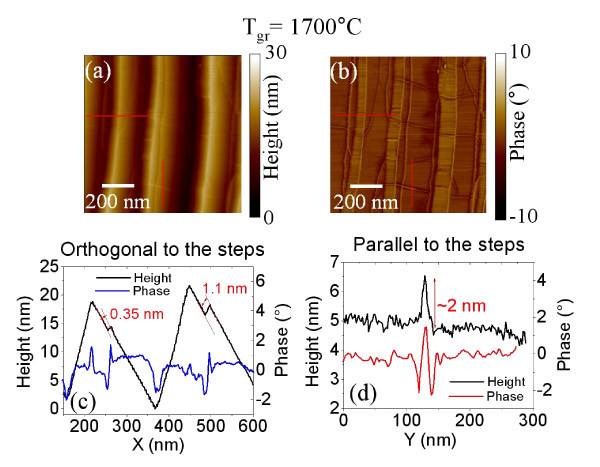
**Details of the morphology and phase maps for the sample annealed at 1700°C**. Morphology (a) and phase (b) maps and two representative linescans taken in the direction orthogonal (c) and parallel (d) to the steps.

The morphological transformation of SiC surface after annealing is described in the following. AFM images of the surface morphology for the samples annealed at 1600, 1700, and 2000°C are reported in Figure 2a,b,c, whereas the corresponding phase maps on the same samples are reported in Figure 2d,e,f. Annealed samples show wide terraces running parallel to the original steps in the virgin sample. An average terrace width of approx. 150 to 200 nm has been estimated for all the annealing temperatures, a significant increase over the small terraces observed for pristine SiC (approx. 30 nm). The estimated RMS roughness for the three samples is approx. 10 nm, which is significantly higher than on the pristine SiC substrate. Such large terraces on annealed samples are the result of the step-bunching commonly observed on off-axis SiC substrates after thermal treatments at temperatures > 1400°C. Interestingly, a network of nanometer wide linear features is superimposed to these large terraces. These features are particularly evident in the phase images of the surfaces. By accurately analyzing the morphology and phase images for the three samples, further insight can be achieved on the nature of these peculiar features. As an example, Figure 3 shows two representative linescans taken in the direction orthogonal (c) and parallel (d) to the steps obtained from on the morphology (a) and phase (b) maps for the sample annealed at 1700°C. In the direction orthogonal to the steps, it is worth noting in the height linescan, some very small steps with nm or sub-nm height are overlapped to the large terraces of the SiC substrate. Each step in the morphology corresponds to the characteristic sequence of a valley and peak in the phase linescan (Figure 3c). It is worth noting that the height of these steps is always a multiple of 0.35 nm, the height value corresponds the interlayer spacing between two stacked graphene planes in HOPG, as typically measured by AFM. As an example, an approx. 0.35-nm and an approx. 1.1-nm high step are indicated in Figure 1c. These step heights can be associated, respectively, to one and three graphene layers over the substrate or stacked over other graphene layers. As reported by other authors, graphene growth on SiC initiates at the terrace step edges of the substrate and continues over the terraces [8].

From the linescan in the direction parallel to the steps (d), it can be observed that the features in the morphology map are some peculiar corrugations with 1 to 2 nm typical height. These features appear on the phase linescan as a peak surrounded by two valleys. It is worth noting that these peculiar corrugations run preferentially in direction orthogonal to the step edges of the substrate.

In order to get further insight on the structural properties of the graphene films grown at the different temperature and to clarify the nature of these peculiar corrugations, HRXTEM analyses were carried out.

**Figure 4:**
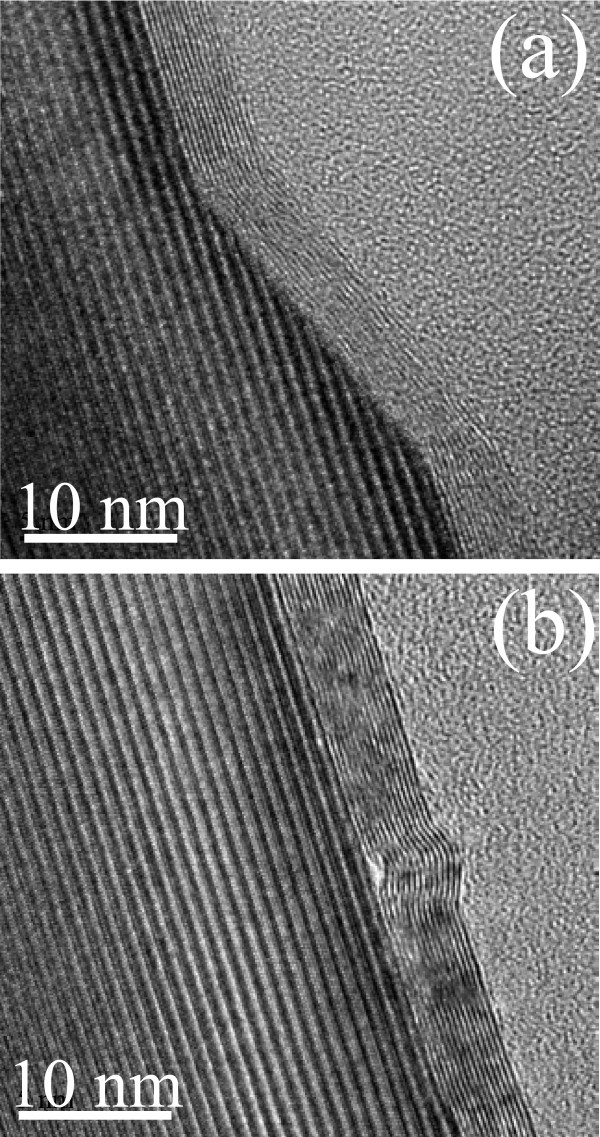
**Representative HRXTEM images**. Sample annealed at 2000°C.

As an example, in Figure 4a,b, two representative HRXTEM images for the sample annealed at 2000°C are reported. Figure 4a shows that a graphene multilayer conformally covers the SiC surface also over the terrace step edges. For each analysis, several HRTEM images have been collected focusing the electron beam on adjacent regions for ~10 μm distances and in all the cases the SiC surface resulted to be covered by FLG.

From Figure 4(b), the structure of one of the peculiar corrugations running orthogonal to the steps is reported. This cross sectional image unambiguously demonstrate that those features are wrinkles in the multilayer graphene film.

These peculiar defects have been observed also by other authors in the case of few layers of graphene grown on the C face [13] or on the Si face [14] of hexagonal SiC, on-axis. However, in that case, wrinkles do not exhibit any preferential orientation with respect to the steps, but form an isotropic mesh-like network on the surface, where wrinkles are interconnected into nodes (typically three wrinkles merge on a node and the angles subtended by the wrinkles are approx. 60 or 120°C [13]). The formation of that mesh-like network of wrinkles was attributed to the release of the compressive strain, which builds up in FLG during the sample cooling due to mismatch between the thermal expansion coefficients of graphene and the SiC substrate [13,15]. In the present case of FLG grown on the Si face of off-axis 4H-SiC, a preferential orientation of the wrinkles orthogonal to the steps is observed. This peculiar effect suggests that compressive strain in FLG is released not only by the formation of parallel wrinkles inside each terrace, but also in an efficient way at step edges. This effect deserves further investigations.

**Figure 5:**
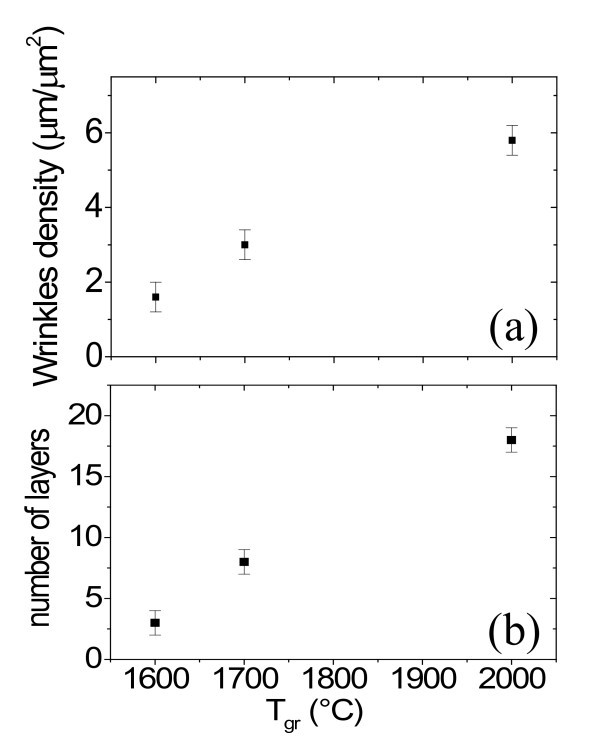
**Increase of the density of wrinkles and of the number of layers as a function of the growth temperature T_gr_**. Average length of the wrinkles per unit area (a). Average number of grown layers (b).

Comparing the morphology and phase images in Figure 2 for the samples annealed at the different temperatures, it is easy to observe that the density of wrinkles increases with temperature. The average length of the wrinkles per unit area (μm/μm^2^) as a function of *T*_gr_, as obtained by a statistical analysis of a large number of AFM images collected each sample, is reported in Figure 5a.

**Figure 6:**
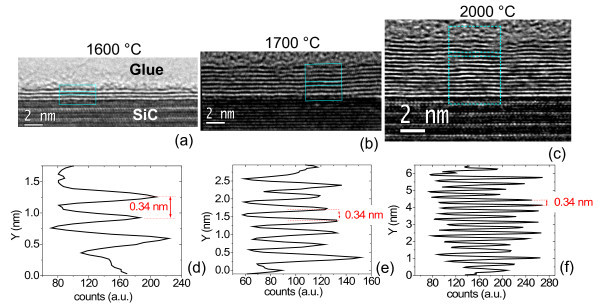
**HRXTEM analyses on the samples annealed at different temperatures**. Images on the samples annealed at 1600 (a), 1700 (b) and 2000°C (c), and corresponding linescans ((d), (e) and (f)), showing 3, 8 and 18 layers grown on the surface of 4H-SiC, respectively.

**Figure 7:**
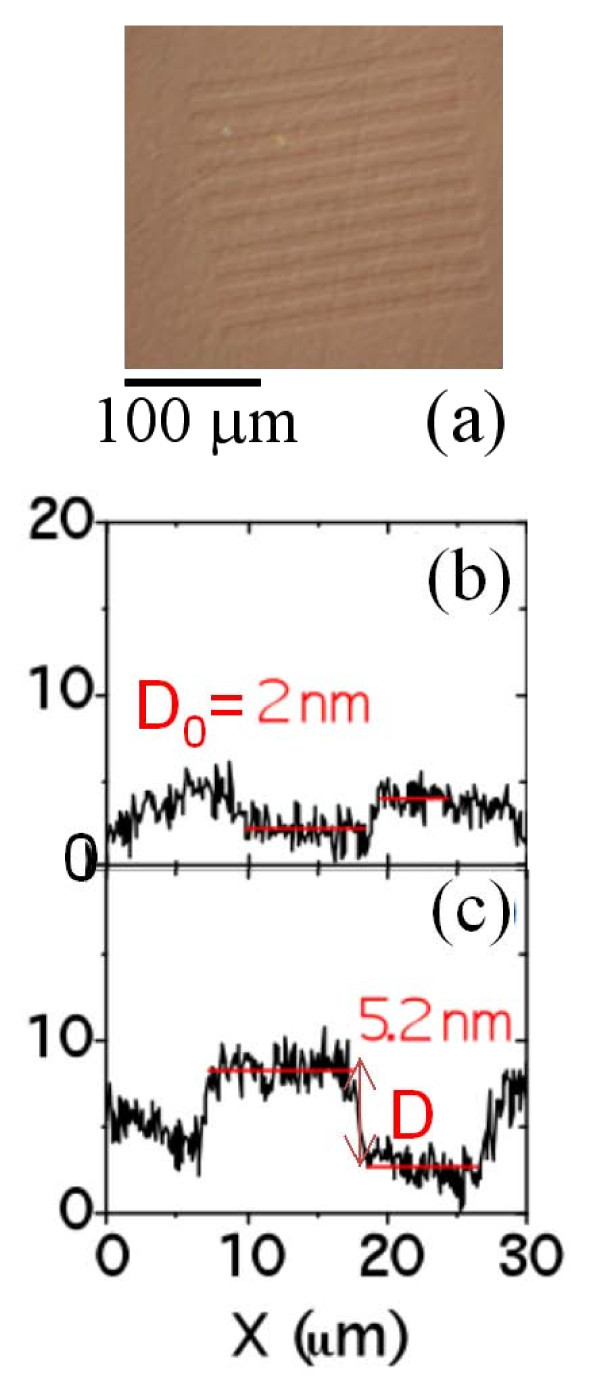
**Optical and AFM images of O_2 _etched striped in graphitized 4H-SiC**. Optical image on the sample annealed at 1700°C (a). AFM height profile taken on a stripe on pristine SiC (b) and on the sample annealed at 1700°C (c), respectively.

In the following, we will study the increase in the average number of graphene layers as a function of the growth temperature. HRXTEM images on the samples annealed at 1600, 1700 and 2000°C are reported in Figure 6a,b and 3c. From the linescans in Figure 3d,e and 3f, the interlayer spacing (~0.34 ± 0.01 nm) as well as the number of graphene layers on the surface of 4H-SiC is determined: 3, 8 and 18 layers can be estimated for the three temperatures. These cross-sectional analyses give a direct measure of the number of grown layers, but only on a very local scale. Lateral variation of the FLG thickness on different sample positions cannot be determined by such a method. To get an estimation of the number of layers at selected surface positions and with higher statistics, t-AFM was used to measure the depth of selectively etched stripes in FLG by O_2 _plasma. This plasma treatment is known to remove efficiently carbonaceous species through a chemical reaction leading to the formation of CO_2_. In Figure 7a, an optical image of the etched stripes in the sample annealed at 1700°C is reported. To obtain an accurate estimation, we checked if the SiC substrate is slightly etched by the used plasma processing. To this aim, a lithographically patterned pristine SiC substrate was simultaneously etched together with the graphitized SiC samples. Figure 7b,c show the height profile taken on a stripe on pristine SiC and on the sample annealed at 1700°C, respectively. From Figure 7b, it is clear that a thickness *t*_0 _~2 nm of SiC is etched during the plasma treatment, due to the physical action of the plasma. This depth must be subtracted while evaluating the number of layers on graphitized SiC. Hence the number of layers can be estimated according to the relation *n *= (*D *- *D*_0_)/*D*_gr_, being *D*_gr _the interlayer separation between two stacked graphene layers (*D*_gr _≈ 0.35 nm). The average number of grown layers as a function of *T*_gr _is reported in Figure 5b, where the error bars represent the standard deviations obtained from a large statistics on the number of layers determined at several sample positions.

## Conclusion

Few-layers of graphene grown on 4H-SiC (0001) 8° off-axis, by annealing in inert gas ambient (Ar) in a wide temperature range (from 1600 to 2000°C) have been structurally characterized by atomic force microscopy and HR-XTEM. For all the considered growth temperatures, FLG conformally covering the 100 to 200-nm wide terraces of the SiC surface have been observed. The formation of wrinkles (approx. 1 to 2 nm high and 10 to 20 nm wide) preferentially oriented in the direction perpendicular to the step edges of the SiC terraces has been observed in the FLG film, as a result of the release of the compressive strain accumulated in FLG during the sample cooling due to the thermal expansion coefficients mismatch between graphene and SiC. This parallel orientation of wrinkles is peculiar of EG grown a vicinal SiC surface, whereas for EG grown on on-axis 4H-SiC an isotropic mesh-like network of wrinkles interconnected into nodes is commonly reported. The observed phenomenon deserves further investigations. Both the density of wrinkles and the number of graphene layers are found to increase almost linearly as a function of the growth temperature in the considered temperature range.

## Abbreviations

CVD: chemical vapor deposition; EG: epitaxial graphene; FLG: few layers of graphene; HR-TEM: high-resolution cross-sectional transmission electron microscopy; HOPG: highly oriented pyrolithic graphite; RMS: root mean square; t-AFM: tapping mode atomic force microscopy

## Competing interests

The authors declare that they have no competing interests.

## Authors' contributions

FG and VR conceived the study. FG coordinated the experiment and participated to the analysis of the data. FG, MR and RY worked on graphene growth. CV and SS carried out the t-AFM measurements and the analysis of the data. CV prepared the sample cross-sections for HR-TEM analyses and CB carried out the HR-TEM measurements. FG and CV wrote the article. All the authors read and approved the manuscript.
